# Development of Predictive Models for Survival among Women with Breast Cancer in Malaysia

**DOI:** 10.3390/ijerph192215335

**Published:** 2022-11-20

**Authors:** Mohd Nasrullah Nik Ab Kadir, Najib Majdi Yaacob, Siti Norbayah Yusof, Imi Sairi Ab Hadi, Kamarul Imran Musa, Seoparjoo Azmel Mohd Isa, Balqis Bahtiar, Farzaana Adam, Maya Mazuwin Yahya, Suhaily Mohd Hairon

**Affiliations:** 1 Department of Community Medicine, School of Medical Sciences, Universiti Sains Malaysia, Kubang Kerian 16150, Kelantan, Malaysia; 2 Biostatistics and Research Methodology Unit, School of Medical Sciences, Universiti Sains Malaysia, Kubang Kerian 16150, Kelantan, Malaysia; 3 Malaysian National Cancer Registry Department, National Cancer Institute, Ministry of Health Malaysia, Putrajaya 62250, Federal Territory of Putrajaya, Malaysia; 4 Breast and Endocrine Surgery Unit, Department of Surgery, Hospital Raja Perempuan Zainab II, Ministry of Health Malaysia, Kota Bharu 15586, Kelantan, Malaysia; 5 Department of Pathology, School of Medical Sciences, Universiti Sains Malaysia, Kubang Kerian 16150, Kelantan, Malaysia; 6 Public Health Division, Penang State Health Department, Ministry of Health Malaysia, Georgetown 10590, Penang, Malaysia; 7 Department of Surgery, School of Medical Sciences, Universiti Sains Malaysia, Kubang Kerian 16150, Kelantan, Malaysia

**Keywords:** breast neoplasm, predictive model, cancer survival, Malaysian women

## Abstract

Prediction of survival probabilities based on models developed by other countries has shown inconsistent findings among Malaysian patients. This study aimed to develop predictive models for survival among women with breast cancer in Malaysia. A retrospective cohort study was conducted involving patients who were diagnosed between 2012 and 2016 in seven breast cancer centres, where their survival status was followed until 31 December 2021. A total of 13 predictors were selected to model five-year survival probabilities by applying Cox proportional hazards (PH), artificial neural networks (ANN), and decision tree (DT) classification analysis. The random-split dataset strategy was used to develop and measure the models’ performance. Among 1006 patients, the majority were Malay, with ductal carcinoma, hormone-sensitive, HER2-negative, at T2-, N1-stage, without metastasis, received surgery and chemotherapy. The estimated five-year survival rate was 60.5% (95% CI: 57.6, 63.6). For Cox PH, the c-index was 0.82 for model derivation and 0.81 for validation. The model was well-calibrated. The Cox PH model outperformed the DT and ANN models in most performance indices, with the Cox PH model having the highest accuracy of 0.841. The accuracies of the DT and ANN models were 0.811 and 0.821, respectively. The Cox PH model is more useful for survival prediction in this study’s setting.

## 1. Introduction

Breast cancer is the most common cancer among women globally and in Malaysia [1,2,3]. Besides overall well-being and physical functioning, overall survival was rated as the most important outcome for Malaysian women with breast cancer [4]. However, the survival rate was lower than in other developed countries such as Japan, Australia and neighbouring Singapore [5].

Survival estimates based on the overall Tumour Nodes Metastasis (TNM) stage at diagnosis alone are useful, but less accurate in communicating survival estimates than the combination of clinical and pathological characteristics [6]. To improve the accuracy, individualised survival based on a prognostic model that incorporated clinical, pathological and biomarkers parameters were developed [6,7,8]. A prognostic model is a prediction model to estimate the probability that a specific event will occur in the future [9].

Breast cancer survival prognostic models were mainly based in the western hemisphere and on developed countries’ patients. The most frequently validated and helpful models are the Nottingham Prognostic Index (NPI), Adjuvant! Online, CancerMath and PREDICT breast cancer [8,10,11,12,13]. However, these tools showed mixed results, and tended to overestimate the survival probability among women with breast cancer in Malaysia [14,15,16].

Furthermore, breast cancer survival studies among Malaysians consistently demonstrated an association between ethnicity and risk of death, necessitating the inclusion of the variable in new predictive models [5,17,18,19]. These studies, however, had several shortcomings, including a lack of generalisability because they were conducted in a single institution [18,20,21]. Meanwhile, population-based cancer registries did not contain vital clinical parameters, as they were mainly used for surveillance, hindering the dataset from being used for predictive modelling [5,17,19].

The stage at diagnosis remained the most important predictor of cancer survival. Other important clinical determinants are histological type and cancer grade. Biomarkers, such as the estrogen receptor (ER), progesterone receptor (PR) and human epidermal growth factor receptor 2 (HER2) became prominent prognostic factors, as new evidence emerged and more women were diagnosed early. Additionally, sociodemographic factors such as ethnicity, marital status and age at diagnosis were shown to alter breast cancer survival probability significantly [22,23]. Based on these prognostic factors, the prognosis for survival is closely related to the type of surgery, chemotherapy and radiotherapy received. The most common predictors used to develop prognostic models are nodal status, tumour size, tumour grade, age at diagnosis, marital status and oestrogen receptor (ER) [10,24]. Therefore, these predictors are used in our study.

The frequently used methods for model development are Cox proportional hazards regression, followed by artificial neural network and decision tree algorithm [10,24]. The latter two supervised machine learning (ML) approaches have emerged as promising techniques to improve prediction accuracy [18,25]. The approaches could identify nonlinear patterns and optimise outcome predictions.

Therefore, this study aimed to develop and validate the five-year survival probability predictive models among women with breast cancer in Malaysia, diagnosed between 2012 and 2016, considering local experience to provide a more precise survival estimation. Subsequently, if the models perform well, a web-based prognostic tool will be developed to enable healthcare providers to communicate disease trajectories with their patients.

## 2. Materials and Methods

### 2.1. Study Area

Malaysia is a multiracial upper middle-income country situated at the centre of the southeast Asian region, with an estimated 32 million residents [26]. We selected seven breast cancer care centres across five regions of Malaysia to better represent our heterogeneous population. 

### 2.2. Study Design and Patients’ Selection

We drew a sample of women who were diagnosed with breast cancer between 1 January 2012 and 31 December 2016 from the Malaysian National Cancer Registry (MNCR) and who were treated at the designated centres. The MNCR is a population-based cancer registry that receives cancer notifications from nationwide medical centres, mortality registries and pathology laboratories [2,5]. 

We retrospectively abstracted the sampled patients’ predictors and survival outcomes information by reviewing individuals’ medical records, as observed longitudinally from the incidence date until 31 December 2021 (the end date of this study). The outcome (i.e., alive or dead status, regardless of the cause) of each patient is updated annually in the MNCR database, as the registry data are linked with the national mortality registry, ensuring almost complete observation of the outcome. We collected the data between September 2021 and February 2022. Data abstraction from patient records was performed by data enumerators who were trained cancer registrars, and by those with medical qualifications.

Our study included Malaysian female citizens aged 18 years old and older with a confirmed histopathological diagnosis of first primary breast cancer (International Classification of Disease for Oncology, 3rd edition [ICD-O-3], topographical codes from C50.1 until C50.9). We excluded cases with date errors (i.e., the diagnosis date was later than the date of death), missing stage information at diagnosis, and unverifiable survival status at five years following diagnosis.

### 2.3. Sample Size Estimation and Sampling Procedure

The sample size was estimated using “pmsampsize”, an R package, to calculate the minimum sample size required to develop a multivariable prediction model with a survival outcome [27]. The minimum sample size was 518 for model development. The calculation was based on 28 anticipated parameters from 13 predictors, an adjusted R-squared value of 0.427 (derived from c-statistic = 0.779) [8], an estimated mean follow-up time of 4.5 years, an overall event rate of 0.37 [5], and 5 years as the time point of interest. 

This study’s analysis employed a random data-splitting strategy with a 7:3 ratio for the derivation and validation datasets. Hence, an additional 222 samples for validation were required. In addition, we considered the possibility of a 50% [28] attrition rate, due to failure to retrieve and review patients’ medical records. Therefore, the final sample size requirement was calculated to be 1480.

We used stratified sampling that was proportionate to the size method, in order to select a sample of breast cancer patients. The strata were divided into five regions in Malaysia, in order to better represent the diverse population and ethnic distribution. Seven centres were selected. Thus, each region was represented by one or two centres. The strata allocations and the number of samples of each region were determined according to the latest cancer registry report [5]. The MNCR database acted as the sampling frame. Subsequently, patients who met the study criteria within each stratum were selected using simple random sampling. 

### 2.4. Study Variables and Data Preprocessing

We selected 13 variables to predict breast cancer survival based on their importance, as mentioned in several studies [8,10,12,18,19,28,29], on anticipated availability in patients’ medical records, and on our interest as experts and researchers. The predictors were age at diagnosis, ethnicity, marital status, histological type, cancer grade, ER, PR and HER2 status; tumour (T), node (N) and metastatic (M) stages; receipt of definitive surgery, chemotherapy and radiotherapy.

Missing information on tumour grade, HER2, ER and PR status were coded as “unknown”. We categorised ethnicity into Malay, Chinese, Indian and other ethnic groups. The histological type was divided into three groups: ductal carcinoma, lobular carcinoma and “others”. We grouped ER and PR status as both ER- and PR-positive, either ER- or PR-positive, both negative of ER and PR and unknown. HER2 status was classified as positive, negative or unknown for those found to be equivocal, not tested and unrecorded. Cancer grades were based on modified Bloom–Richardson histologic grading. T-, N-, and M-stages were determined by treating physicians using the American Joint Committee on Cancer, AJCC 7th edition Cancer Staging. Treatment received, such as surgery, chemotherapy and radiotherapy, were coded as “Yes” or otherwise as “No”.

For the ANN model, we performed data preprocessing for the predictors’ variables. The continuous variables (i.e., age at diagnosis) were transformed using min-max scaling, in order to constrain values between zero and one. Meanwhile, the categorical variables were converted into dummy variables.

### 2.5. Analysis Method

We input the data into a Microsoft Excel document and exported it to R software for analysis. A random-split strategy with a 7:3 ratio for derivation “training” datasets and validation “testing” datasets was employed. Descriptive statistics were used to summarise the socio-demographic and clinical characteristics of the patients. Numerical data were presented as means and standard deviations (SD), or median and interquartile range (IQR), according to normality distribution. Categorical data were presented as frequencies (*n*) and percentages (%). 

The outcome variable or event of interest was death, regardless of the cause. Patients who did not experience the event were considered censored at the latest follow-up encounters or at the end of the study (31 December 2021). Survival time refers to the duration (in years) from the confirmed histopathological diagnosis dates, to the event or last follow-up/end of the study for censored observations. Overall survival (OS) was calculated using the Kaplan–Maier method. 

We used three modelling approaches for analysis, which were multivariable Cox proportional hazard regression (Cox PH), decision tree classification analysis (DT) and artificial neural networks (ANN). For multivariable Cox PH, we employed the R packages “survival”, “survminer”, and “pec”. We included all 13 predictors to develop the models and predict the survival probabilities. We assessed any two-way interactions between T-, N-, M-stage and M-stage with treatment options due to potential interactions and different responses, to treatment based on clinical knowledge. The proportional hazards assumptions were assessed graphically, using tests of Schoenfeld residuals. The adjusted hazard ratio (HR) of each predictor was presented. All *p*-values were two-sided, and *p* < 0.05 was considered statistically significant. 

To explore the potential prediction accuracy of the machine learning classifier, we employed DT and ANN algorithms, which have frequently been used to train models for the prediction of five-year survival status among women with breast cancer [10,24]. For these models, the outcome was dichotomous: either alive at five years after diagnosis, or dead within five years. For the DT model, the R packages used for analysis and visualisation of the decision tree were “rpart” and “rpart.plot”. Default hyperparameter values (minsplit = 20 and minbucket = 7) were used. For ANN, the multi-layer perceptron model consisted of sigmoid activation functions, with one hidden layer having two hidden nodes. We used the R package “neuralnet” for analysis. 

To facilitate an acceptable comparison between binary survival models (DT and ANN) and the time-to-event Cox PH model, we obtained the predicted five-year survival probability by fitting the model that was developed using the derivation dataset, and compared it with the observed survival status at five years to validate the models’ performance. We measured the models’ performance [10,24,30] in terms of calibration (predicted survival versus observed survival), discriminant (area under the ROC), clinical utility (accuracy, sensitivity, specificity, positive predictive value (PPV) and negative predictive value (NPV)), as well as precision, recall and F1-Score. In addition, we computed the concordance index (c-index) [31], the calibration plot [32] and the integrated Brier score [30] for the Cox PH model.

### 2.6. Ethics Statement

We obtained ethical approval from the Medical Research and Ethics Committee, Ministry of Health Malaysia (NMRR-21-37-57989 (IIR)) and the Human Research and Ethics Committee, Universiti Sains Malaysia (USM/JEPeM/21010112). Permission to use non-identifying patient records was obtained from the data custodians of the participating centres’ directors at the Ministry of Health Malaysia. We ensured the confidentiality of the patient’s data by restricting access only to the researchers. These data were used under agreement for the current study, and are not publicly available without the explicit permission of the Director General, Ministry of Health Malaysia.

## 3. Results

Out of 1480 samples, we retrieved and reviewed 1073 medical records. A total of 64 patients out of 1073 were excluded for the following reasons: diagnosis of other cancers (*n* = 2), missing stage at diagnosis information (*n* = 43), and unverifiable survival status at five years after diagnosis (*n* = 19). Among the 1006 patients included in the analysis, 485 (48.2%) succumbed to breast cancer or other causes within the study period. The median (IQR) follow-up time was 5.5 (2.7–6.8) years. We randomly split the patients into groups, derivation (*n* = 704) and validation datasets (*n* = 302).

### 3.1. Descriptive Statistics

Our patients’ mean (SD) age at diagnosis was 53.1 (11.7). They were mostly Malay (55.8%), had ductal carcinoma (89.4%), were hormone-sensitive (49.4% both ER- and PR-positive), HER2 negative (49.2%), at T2- (40.7%), N1-stage (37.3%), had no metastasis (73.7%), and received early definitive surgery (80.5%) and chemotherapy (62.2%). Detailed descriptive statistics are presented in Table 1.

### 3.2. Five-Year Survival

**Figure 1:**
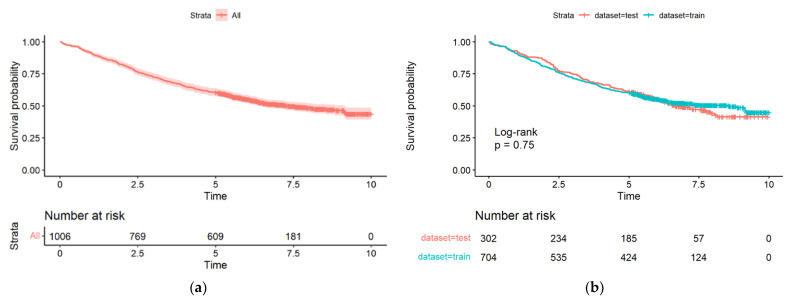
Kaplan–Maier survival curves for (**a**) all patients; (**b**) between derivation “train” dataset and validation “test” dataset.

The estimated five-year survival for all data was 60.5% (95% CI: 57.6, 63.6). The five-year survival status (Table 1) and overall survival (Figure 1 and Table 2) were not statistically significant between derivation and validation datasets, ensuring reliable validation findings.

### 3.3. Cox Regression Analysis 

Malaysian Indian women had a higher risk of mortality compared to Malay women. Women with lobular carcinoma had a higher risk of death than women with the more common ductal carcinoma. As expected, higher grades, T-, N- and M-stages are associated with an increased risk of death. There was a decreased risk of death among the cohort who received any treatment. Compared to those with positive ER and PR status, patients with negative ER and PR status had an increased risk of death, albeit not reaching a statistically significant level. The findings showed statistically non-significant differences in mortality risk between different ages, marital status, and HER2 status. Results of the multivariable Cox proportional hazards regression model to predict death are shown in Table 3.

### 3.4. Decision Tree Classification Analysis

**Figure 2:**
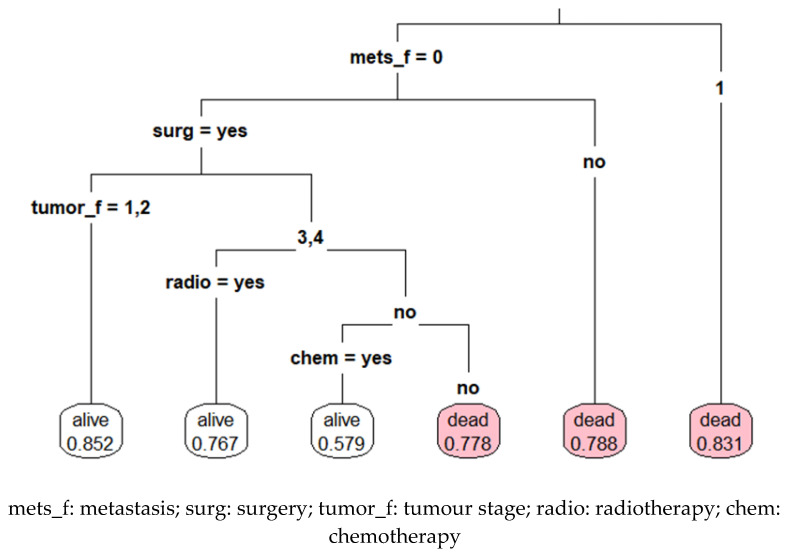
Decision tree classification analysis to predict death at five-year time point among patients with breast cancer (*n* = 704). The leaf at the bottom of the tree refers to the probability of outcomes. The white leaf showed the probability of being alive, whereas the pink leaf showed the probability of being dead.

The decision tree analysis generated a visual representative algorithm tree, as shown in Figure 2. The variables that were found to be the decision node before the terminal node for the algorithm were the presence of metastasis, receipt of surgery status, tumour stage at diagnosis, radiotherapy status and chemotherapy status. In general, women with a lower stage at diagnosis who received any treatment had a higher probability of survival. Figure 1 shows patients without metastasis who received surgery at T-stage 1 and 2 had an 85.2% five-year survival probability. Meanwhile, those with metastasis had merely a 16.9% five-year survival probability (i.e., 83.1% probability of death). 

### 3.5. Model Validation and Performance Comparison

**Figure 3:**
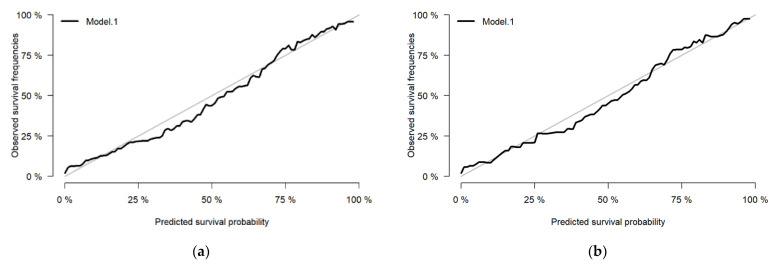
The calibration plots show a well-calibrated Cox PH model for (**a**) the derivation dataset and (**b**) the validation dataset between observed and predicted survival probabilities. Calibration plots (black line) along the 45-degree line (grey line) indicate a perfect calibration model in which the predicted survival probabilities are identical to the observed survival outcomes.

For Cox PH, the c-index for our derivation model was 0.82 (standard error, SE: 0.01), and the c-index for the validation dataset was 0.81 (SE: 0.02). The integrated Brier score was 0.115 for the development dataset, and 0.122 for the validation dataset. The calibration plot of the observed survival versus the predicted survival was satisfactory, as shown in Figure 3.

The three models’ performance indices (Table 2) were good and comparable in the derivation datasets. All predicted survivals were approximately similar to the observed survivals. For validation datasets, the Cox PH model outperformed the DT and ANN models in terms of AUC, accuracy, sensitivity, NPV, recall and F1-Score. Meanwhile, the DT model scored highest in specificity, PPV and precision value.

## 4. Discussion

In this study, we developed predictive models based on our patients’ experiences using well-known clinical and socio-demographic parameters, in order to predict survival probability. We found all three models to have good performance, with Cox PH having slightly better performance. Unlike the previous study that was based on a single institution located within the highly urbanised national capital [18], our model is likely to be more generalisable among women with breast cancer in Malaysia, as we collected the data across several breast cancer referral centres from each corner of the country. For instance, the aforementioned model disproportionately included lower metastatic breast cancer, and higher numbers of Chinese patients [18]. 

We included local ethnic groups as a predictor to reflect the differential survival risk [5,17,19], which was not included by the previous prognostic tool [33,34]. Furthermore, local ethnic groups or races were considered to be valuable predictors in developing a new prognostic model in Taiwan and New Zealand [12,13]. Existing well-known prognostic tools, such as PREDICT, CancerMath and NPI, showed some limitations when applied to Malaysian women, possibly due to the difference in population case mix. Our patients tended to have unfavourable clinical presentations at diagnosis, in comparison to the cohort that was used to develop the previous model [15,35]. 

A breast cancer survival study in a northern state within peninsular Malaysia showed a remarkably favourable five-year survival rate of 72.9% (*n* = 2166) [36]. Meanwhile, the five-year survival rate found in our study is approximately similar to the previously analysed nationwide study of 61.9% (*n* = 17,009); thus, this suggests there are regional differences in cancer prognoses that necessitate the inclusion of centres across different regions within Malaysia.

For the Cox PH model, we found the Indian ethnic group contributed substantially to the prediction of survival of women with breast cancer in Malaysia. Malaysia comprises three major ethnic groups: Malay, Chinese and Indian, with distinct socio-economic levels and culture-related health-seeking behaviours. Indian and Malay ethnic groups are generally associated with lower socio-economic levels. They have lower survival rates. In addition to the potential biological cause, they are more likely to present a later stage at diagnosis, seek alternative treatment and face financial constraints [19,29,37,38,39,40]. Besides ethnicity, histological type, tumour grade, TNM stage and treatment were associated with mortality, which was consistent with previously published studies [8,12,13,19]. A lower stage at diagnosis reduces the risk of death. This statement further strengthens the importance of early detection of breast cancer with mammogram screening in an eligible population [41,42]. The model showed robust internal validity. The value of the Brier score in our study was less than 0.25, indicating good predictive ability. The c-index of more than 0.8 from internal validation showed strong discriminatory power [43], which is comparable to developed models in the United States [8], New Zealand [12] and Taiwan [13]. The calibration assessment indicated good agreement between predicted and observed survival. 

For the DT model, our finding is similar to the previous study in Malaysia [18], which stated that cancer stage, tumour size, positive lymph nodes and primary type of treatment were among the most important variables. We used various measures of performance indices to compare the three models. In general, survival prediction in the validation dataset improved when we employed the Cox PH model. In this study, we compared the models’ performances on the basis of the five-year survival binary outcome. A study that compared Cox PH with machine learning classifiers found that the ANN model more accurate in predicting five-year mortality among breast cancer patients in Taiwan [28]. Meanwhile, a machine learning study among breast cancer patients in Malaysia showed that all six algorithms, including the DT and ANN models, performed well with non-substantial differences in terms of model accuracies [18]. Several systematic reviews [10,24,44] concluded that machine learning approaches do not always result in better prediction estimation and clinical validity. Time-to-event machine learning approaches have been developed [45,46]. However, these approaches generally come with concerns regarding the interpretability, transparency and robustness of models’ validation [47,48]. 

The strength of our study is that we used recently available data from the MNCR database, which is linked to the national mortality registry, in order to ensure that the survival outcome data were complete. Secondly, we sampled a cohort across multiple centres that was spread across the nation, in order to better represent our population and minimise referral bias or biases reflecting the practices of a single institution and care provider. Thirdly, we included only common prognostic factors that could be easily applied in future practices.

The limitations of our study are inherent to retrospective observational record review data with a degree of missing data. We minimised the missing data during data collection by including prognostic factors that are usually recorded in medical records. In addition, four out of seven centres chosen were implementing electronic medical records. Consequently, we could not include predictors such as socio-economic status, targeted therapy and hormonal therapy. To minimise misclassification errors, we recruited trained cancer registrars and those with medical backgrounds to collect the data. Another limitation is related to the model comparison. The DT and ANN models were based on binary five-year survival outcomes, whereas the Cox PH model is a time-to-event analysis. Although the models were compared at an exact time, and we only included patients with a verifiable survival status at five years, the risk of bias cannot be excluded. Despite these limitations, all three models were found to have comparable performance.

Our study used a similar dataset for validation. Further evaluation using an independent dataset would be valuable to ensure transportability, if there are changes in screening, diagnosis and treatment practices across different centres and future periods. We recommend that subsequent prognostic models use prospectively collected cancer data with a larger dataset, as a more comprehensive electronic clinical cancer database is being implemented in our country. Our future studies will involve designing a web-based prognostic tool for easier accessibility of healthcare providers when dealing with newly diagnosed breast cancer patients and comparison analysis of our model’s predictive performance with an existing tool such as the PREDICT breast cancer tool.

## 5. Conclusions

We developed predictive survival models among women with breast cancer in Malaysia, and all models showed good performance. The Cox PH model had slightly better overall performance. The model could potentially aid healthcare providers in estimating individualised patient outcomes. Our research provides a promising benefit for the community of women with breast cancer as they seek more accurate expectations of their health trajectory.

## Figures and Tables

**Table 1:** Descriptive characteristics of women with breast cancer included for analysis, 2012–2016 (*n* = 1006).

Characteristics	All Patients, *n* (%)	Derivation Dataset, *n* (%)	Validation Dataset, *n* (%)	*p*-Value ^1^
Number of patients (*n*)	1006	704	302	
Follow-up time (years)				
median (IQR)	5.5 (2.7–6.8)	5.5 (2.6–6.7)	5.5 (2.9–6.8)	
Number of total deaths	485 (48.2)	334 (47.4)	151 (50.0)	0.457
**Five-year survival status**				
Alive Dead	609 (60.5) 397 (39.5)	424 (60.2) 280 (39.8)	185 (61.3) 117 (38.7)	0.759
**Age**, mean (SD) in years	53.1 (11.7)	53.2 (11.8)	52.9 (11.3)	0.701
**Ethnicity**				
Malay Chinese Indian Others	561 (55.8) 316 (31.4) 74 (7.4) 55 (5.5)	392 (55.7) 215 (30.5) 50 (7.1) 47 (6.7)	169 (56.0) 101 (33.4) 24 (7.9) 8 (2.6)	0.070
**Marital status**				
Married Not married	788 (78.3) 218 (21.7)	554 (78.7) 150 (21.3)	234 (77.5) 68 (22.5)	0.669
**Histological type**				
Ductal carcinoma Lobular carcinoma Others	899 (89.4) 46 (4.6) 61 (6.1)	624 (88.6) 37 (5.3) 43 (6.1)	275 (91.1) 9 (3.0) 18 (6.0)	0.281
**Grade**				
Well-differentiated Moderately differentiated Poorly differentiated Unknown	174 (17.3) 300 (29.8) 217 (21.6) 315 (31.3)	123 (17.5) 209 (29.7) 155 (22.0) 217 (30.8)	51 (16.9) 91 (30.1) 62 (20.5) 98 (32.5)	0.930
**ER and PR status**				
Both ER- and PR-positive Either ER- or PR-positive Both negative Unknown	497 (49.4) 96 (9.5) 277 (27.5) 136 (13.5)	343 (48.7) 75 (10.7) 201 (28.6) 85 (12.1)	154 (51.0) 21 (7.0) 76 (25.2) 51 (16.9)	0.051
**HER2 status**				
Positive Negative Unknown	228 (22.7) 495 (49.2) 283 (28.1)	163 (23.2) 350 (49.7) 191 (27.1)	65 (21.5) 145 (48.0) 92 (30.5)	0.547
**Tumour (T) stage**				
T1 T2 T3 T4	176 (17.5) 409 (40.7) 174 (17.3) 247 (24.6)	123 (17.5) 284 (40.3) 114 (16.2) 183 (26.0)	53 (17.5) 125 (41.4) 60 (19.9) 64 (21.2)	0.297
**Node (N) stage**				
N0 N1 N2 N3	363 (36.1) 375 (37.3) 178 (17.7) 90 (8.9)	259 (36.8) 256 (36.4) 123 (17.5) 66 (9.4)	104 (34.4) 119 (39.4) 55 (18.2) 24 (7.9)	0.707
**Metastasis (M) stage**				
M0 M1	741 (73.7) 265 (26.3)	521 (74.0) 183 (26.0)	220 (72.8) 82 (27.2)	0.702
**Surgery**				
No Yes	196 (19.5) 810 (80.5)	136 (19.3) 568 (80.7)	60 (19.9) 242 (80.1)	0.840
**Chemotherapy**				
No Yes	380 (37.8) 626 (62.2)	270 (38.4) 434 (61.6)	110 (36.4) 192 (63.6)	0.563
**Radiotherapy**				
No Yes	528 (52.5) 478 (47.5)	364 (51.7) 340 (48.3)	164 (54.3) 138 (45.7)	0.449

^1^ Pearson’s chi-squared test except for age (Welch’s two-sample *t*-test); ER, oestrogen receptor; PR, progesterone receptor; HER2, human epidermal growth factor receptor 2.

**Table 2:** Performance indices of the Cox proportional hazard (PH) regression models, decision tree (DT) analysis and artificial neural network (ANN) for predicting five-year survival of breast cancer patients.

Performance Indices	Derivation Dataset (*n* = 704)			Validation Dataset (*n* = 302)		
	Cox PH	DT	ANN	Cox PH	DT	ANN
Observed OS ^1^ (%)	60.2 (95% CI: 56.7, 64.0)			61.3 (95% CI: 56.0, 67.0)		
Predicted OS ^2^ (%)	59.8	60.2	60.5	60.1	60.5	62.0
AUC	0.886	0.826	0.911	0.891	0.839	0.877
Accuracy	0.820	0.824	0.845	0.841	0.811	0.821
Sensitivity	0.915	0.896	0.946	0.941	0.849	0.898
Specificity	0.675	0.711	0.693	0.684	0.732	0.660
PPV	0.810	0.824	0.823	0.825	0.870	0.847
NPV	0.840	0.819	0.894	0.879	0.696	0.753
Precision	0.810	0.824	0.823	0.825	0.870	0.850
Recall	0.915	0.896	0.946	0.941	0.849	0.898
F1-score	0.859	0.859	0.880	0.879	0.859	0.870

^1^ Kaplan–Maier estimate; ^2^ mean; OS: overall survival; AUC: area under ROC (receiver operating characteristics) curve; PPV: positive predictive value; NPV: negative predictive value.

**Table 3:** Multivariable Cox proportional hazards regression model to predict death among women with breast cancer (*n* = 704).

Variables		*b*	Adjusted HR (95% CI)	*p*-Value
**Age (year)**		−0.005	0.99 (0.98, 1.00)	0.274
**Ethnicity**				
	Malay Chinese Indian Others	−0.214 0.571 0.019	1 0.81 (0.61, 1.00) 1.77 (1.19, 2.63) 1.02 (0.64, 1.62)	0.131 0.005 0.934
**Marital status**				
	Not married Married	0.085	1 1.09 (0.82, 1.45)	0.554
**Histological type**				
	Ductal carcinoma Lobular carcinoma Others	0.490 0.348	1 1.63 (1.05, 2.53) 1.41 (0.80, 2.51)	0.028 0.233
**Grade**				
	Well-differentiated (Grade 1) Moderately differentiated (Grade 2) Poorly differentiated (Grade 3) Unknown	0.610 0.845 0.695	1 1.84 (1.15, 2.94) 2.33 (1.43, 3.78) 2.00 (1.05, 3.84)	0.010 < 0.001 0.036
**ER and PR status**				
	Both positive Either ER or PR positive Both negative Unknown	0.255 0.262 −0.110	1 1.29 (0.88, 1.90) 1.30 (1.00, 1.69) 0.90 (0.52, 1.54)	0.194 0.052 0.690
**HER2 status**				
	Negative Positive Unknown	−0.0003 0.262	1 1.00 (0.75, 1.34) 1.30 (0.96, 1.76)	0.998 0.089
**Tumour (T) stage**				
	T1 T2 T3 T4	0.895 1.223 1.230	1 2.45 (1.43, 4.18) 3.40 (1.93, 6.00) 3.42 (1.95, 6.00)	0.001 < 0.001 < 0.001
**Node (N) stage**				
	N0 N1 N2 N3	0.549 0.730 1.119	1 1.73 (1.24, 2.40) 2.07 (1.43, 3.02) 3.06 (2.02, 4.65)	0.001 < 0.001 < 0.001
**Metastasis (M) stage**				
	M0 M1	0.953	1 2.60 (1.93, 3.50)	< 0.001
**Surgery**				
	No Yes	−0.703	1 0.49 (0.28, 0.87)	0.015
**Chemotherapy**				
	No Yes	−0.535	1 0.59 (0.44, 0.79)	< 0.001
**Radiotherapy**				
	No Yes	−0.353	1 0.70 (0.51, 0.96)	0.026

ER, oestrogen receptor; PR, progesterone receptor; HER2, human epidermal growth factor receptor 2; b, regression coefficient; HR, hazard ratio; CI, confidence interval; no two-way interaction between T-, N-, M-stage; and M-stage with treatments (surgery, chemotherapy and radiotherapy). The proportional hazards showed that all predictors met the criteria except for histological type, ER and PR status, HER2 and radiotherapy. Further examinations of the Schoenfeld residual plots for these four variables were acceptable up to five years after diagnosis.

## Data Availability

The data that support the findings are available from the authors, but restrictions apply to the availability of these data. These data were used under agreement for the current study, and are not publicly available. Data are, however, available from the authors, but only with the explicit permission of the Director General, Ministry of Health Malaysia.
